# Risk of esophageal and gastric adenocarcinoma in men receiving androgen deprivation therapy for prostate cancer

**DOI:** 10.1038/s41598-021-92347-0

**Published:** 2021-06-29

**Authors:** Richard Shore, Jingru Yu, Weimin Ye, Jesper Lagergren, Martin Rutegård, Olof Akre, Pär Stattin, Mats Lindblad

**Affiliations:** 1 Department of Clinical Science, Intervention and Technology (CLINTEC), Karolinska Institutet and Function Perioperative Medicine and Intensive Care, Karolinska University Hospital, Stockholm, Sweden; 2 Department of Medical Epidemiology and Biostatistics (MEB), Karolinska Institutet, Stockholm, Sweden; 3 Department of Molecular Medicine and Surgery, Karolinska Institutet and Karolinska University Hospital, Stockholm, Sweden; 4 School of Cancer and Pharmaceutical Sciences, King’s College London, and Guy’s and St Thomas’ NHS Foundation Trust, London, UK; 5 Department of Surgical and Perioperative Sciences, Surgery, Umeå University, Umeå, Sweden; 6 Wallenberg Centre for Molecular Medicine, Umeå University, Umeå, Sweden; 7 Department of Molecular Medicine and Surgery, Karolinska Institutet, Stockholm, Sweden; 8 Department of Pelvic Cancer, Karolinska University Hospital, Stockholm, Sweden; 9 Department of Surgical Sciences, Uppsala University, Uppsala, Sweden

**Keywords:** Gastric cancer, Oesophageal cancer

## Abstract

The aim of this study was to explore the male predominance in esophageal and gastric adenocarcinoma by evaluating the preventive potential of androgen deprivation therapy (ADT). This matched cohort study was based on a national Swedish database of prostate cancer patients in 2006–2013. Prostate cancer patients receiving ADT were the exposed group. Prostate cancer-free men from the general population were randomly selected and matched to the index case by birth year and county of residence, forming the unexposed control group. The participants were followed until a diagnosis of esophageal or gastric cancer, death, emigration, or end of the study period. The risk of esophageal adenocarcinoma, cardia gastric adenocarcinoma, non-cardia gastric adenocarcinoma, and esophageal squamous-cell carcinoma among ADT-exposed compared to unexposed was calculated by multivariable Cox proportional hazard regression. The hazard ratios (HRs) and 95% confidence intervals (CIs) were adjusted for confounders. There was a risk reduction of non-cardia gastric adenocarcinoma among ADT-users compared to non-users (HR 0.49 [95% CI 0.24–0.98]). No such decreased risk was found for esophageal adenocarcinoma (HR 1.17 [95% CI 0.60–2.32]), cardia gastric adenocarcinoma (HR 0.99 [95% CI 0.40–2.46]), or esophageal squamous cell carcinoma (HR 0.99 [95% CI 0.31–3.13]). This study indicates that androgen deprivation therapy decreases the risk of non-cardia gastric adenocarcinoma, while no decreased risk was found for esophageal adenocarcinoma, cardia gastric adenocarcinoma, or esophageal squamous-cell carcinoma.

## Introduction

Esophageal adenocarcinoma (EAC) is increasing in incidence^1^. Established risk factors include gastroesophageal reflux, obesity^2,3^ and tobacco smoking^4^, while protective factors include *Helicobacter pylori-*infection^5^ and a diet rich in fruit and vegetables^6^. There is an unexplained and strong male predominance in the incidence of EAC with a male:female ratio of 3–9:1^1,7^. Esophageal squamous-cell carcinoma (ESCC) is the most common histological type of esophageal cancer globally^1^ and its incidence has remained fairly stable or decreased in most regions. Risk factors include tobacco smoking, alcohol overconsumption, and intake of red meat and very hot beverages, whereas consumption of fruit and vegetables are protective. The male:female ratio in ESCC is 1–8:1, which is largely explained by sex differences in the prevalence of tobacco smoking and alcohol overconsumption^8,9^. Gastric adenocarcinoma (GAC) is often divided into cardia and non-cardia sub-sites because of differences in etiology and incidence patterns. The past decades have witnessed a decrease in the incidence of non-cardia GAC^10^, whereas cardia GAC has become increasingly common with risk factors and a male predominance similar to EAC^11^. The decrease in the incidence of non-cardia GAC is probably mainly due to the decrease in the prevalence of *Helicobacter pylori*, the main risk factor^12^, but also better food preservation and an increased intake of fruit and vegetables^13^. There is an unexplained male predominance also in the incidence of non-cardia GAC (2–3:1), independent of region, ethnicity and local incidence rates^14^.

In contrast to ESCC, the male predominance seen in EAC and GAC cannot be explained by known risk factors because the sex distribution of risk factors is similar and the strengths of associations are similar between men and women^15^. Differences in the exposures to sex hormones such as androgens and estrogens may play a part in the observed sex difference in incidence of these adenocarcinomas and has been evaluated in several studies, including those examining androgen deprivation therapy (ADT), anti-estrogen therapy, hormone replacement therapy (HRT), as well as sex hormonal and reproductive factors^9,16^. The results are partly conflicting, but on average women seem to be 10–15 years older at diagnosis of gastric cancer than men^17^. The sex ratio in EAC and cardia GAC could be due to sex hormonal factors unrelated to menopause, whereas the sex ratio in non-cardia GAC could be linked to a protective effect of the premenopausal female sex hormonal milieu^18^.

This study aimed to test the hypothesis that androgens increase the risk of EAC, cardia GAC, and non-cardia GAC. ESCC was mainly included as a comparison outcome. We analyzed a nationwide cohort of men with prostate cancer who were exposed to ADT and compared them to a matched cohort of unexposed and prostate cancer-free men.

## Methods

### Data sources

To collect study data, we used the Prostate Cancer data Base Sweden (PCBaSe) version 3.0, which contains information on cancer characteristics and primary treatment from the National Prostate Cancer Register (NPCR) in Sweden^19^. The NPCR covers 98% of all diagnosed cases of prostate cancer compared to the Swedish Cancer Register, became nationwide in 1998^19–21^, and includes information such as age at diagnosis, date of diagnosis, and primary treatment (prostatectomy, radiotherapy, deferred treatment [i.e., watchful waiting or active surveillance] and primary ADT). In PCBaSe 3.0, a comparison cohort of men without prostate cancer has been created by selecting five prostate cancer-free men in a randomized fashion from the Swedish Register of the Total Population, matched to each of the index cases by birth year (attained age) and county of residence^19^. To obtain information on migration, death dates, marital status and educational level, the men with prostate cancer in the NPCR as well as the comparison cohort have been linked to relevant national health data registers and demographic databases: data on migration was obtained from the Register of the Total Population whereas death dates were obtained from the Cause of Death Register and marital status and educational level from the Longitudinal integration database for health insurance and labor market studies^22–24^. Data on drug use was linked to PCBaSe 3.0 from the Swedish Prescribed Drug Register^25^, which contains data on all prescribed drugs in Sweden since its inception on July 1, 2005, forming the PCBaSe^traject^ database.

### Study design

This was a matched cohort study employing the PCBaSe^traject^ database that includes men diagnosed with prostate cancer and information regarding their complete treatment trajectory. Prostate cancer patients receiving ADT in the form of anti-androgens, gonadotropin-releasing hormone (GnRH) analogues (GnRH as well as GnRH + flare protection by use of anti-androgens during a restricted time, usually 1 month), orchiectomy, total androgen blockade with GnRH plus anti-androgens continuously or placed on watchful waiting and put on ADT at a later date were included in the cohort as exposed (ADT group), while prostate cancer patients that did not receive ADT were excluded from the study. We used the comparison cohort of men without prostate cancer diagnosis or ADT described above as the unexposed group. Men with other primary cancers (excluding non-melanoma skin cancer) prior to the diagnosis of prostate cancer or before the start of the study period were excluded. Entry time in the study was set from January 1, 2006 to December 31, 2012. Due to the later start date of the Prescribed Drug Register (1 July 2005), PCBaSe^traject^ requires use of left-truncation in all time-to-event analysis^19^. We wanted to identify incident use of ADT and to do this we needed a wash-out period of 6 months in order to identify men who started ADT as opposed to men who had used it for a long period with an unknown start date (before the inception of the Prescribed Drug Register). Follow-up was started 1 year after start of ADT and was set from January 1, 2007 to December 31, 2013, to allow for a latency time of at least 1 year for cancer development. Importantly, exposure in the cohort was defined as ADT exposed or not. We did not consider the time of prostate cancer diagnosis, since all controls were prostate cancer-free and did not receive any ADT during follow-up, while all cases were prostate cancer patients and received different forms of ADT. Men were followed until either a diagnosis of EAC, cardia GAC, non-cardia GAC, or ESSC, or death, emigration, or end of the study period December 31, 2013, whichever occurred first. Ascertainment of cancer cases was obtained by linkage with the Swedish Cancer Register which includes almost all (98%) incident cases of EAC, cardia GAC, non-cardia GAC, and ESSC^26,27^.

### Statistical analysis

The continuous variables of the study population were presented as means with standard deviations. The categorical variables were presented as frequencies with percentages. The scaled Schoenfeld residuals showed no violation of the proportional hazard assumption. Fisher’s exact test was used for testing differences of distributions of categorical variables in subgroups. To estimate the relative risk of esophageal or gastric cancer among men with ADT exposure, as compared to men without ADT, multivariable Cox proportional hazard regression was applied to calculate hazard ratios (HRs) with 95% confidence intervals (CIs), adjusted for marital status and educational level. Because the controls were individually matched to the cases by attained age and county of residence, these variables were not included in the model. A complete case analysis was performed, because the amount of missing was considered negligible. Stratified analyses were conducted for anti-androgens, GnRH, GnRH + flare, orchiectomy and total androgen blockade. P values < 0.05 were considered statistically significant. A sensitivity analysis pooled EAC and cardia GAC data because of their similar etiology and sex ratio. All statistical analyses were performed using the statistical software Stata (version 12.1; Stata Corporation, College Station, TX, USA).

### Ethics approval

This study was performed in line with the principles of the Declaration of Helsinki. The study was approved by the Ethical Review Board in Stockholm, Sweden (DNR: 2009/1196-31/1) and Umeå, Sweden (DNR: 2015-219-32).

### Consent to participate

Not applicable since all results were yielded in the analysis of anonymized data.

## Results

### Participants

**Figure 1:**
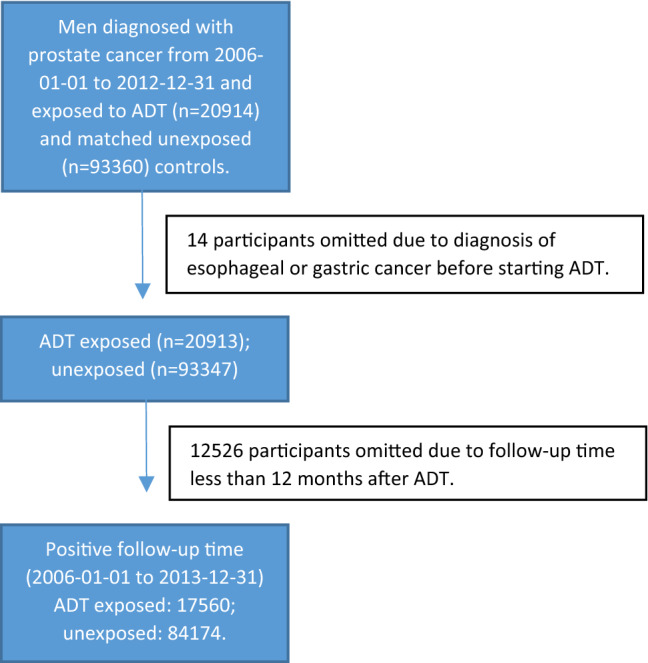
Flowchart of study cohort formation. Prostate cancer patients diagnosed between 2006-01-01 and 2012-12-31 in PCBaSe^traject^ exposed to androgen deprivation therapy (ADT) and their 1:5 matched controls were entered into the cohort. This was a total of 20,914 exposed and 93,360 unexposed participants. Firstly, 14 participants with a diagnosis of esophageal or gastric cancer prior to starting ADT were omitted. Finally, 12,526 participants were omitted due to a follow-up time of less than 12 months. The resulting cohort of eligible individuals for analysis was a total of 101,734 participants with 17,560 ADT exposed, PC cases and 84,174 non-ADT exposed, cancer free controls.

The study identified 20,914 ADT exposed cases with a first diagnosis of prostate cancer and 93,360 ADT unexposed matched controls. After excluding 14 participants with a diagnosis of esophageal or gastric cancer prior to starting ADT and 12,526 participants with a follow-up time of less than 12 months, 17,560 ADT exposed cases and 84,174 unexposed controls remained for final analysis (Fig. 1). The total follow-up time in the cohort was 283,022 person-years (41,569 person-years for cases and 241,453 person-years for controls) and the median follow-up time was 4.3 years (interquartile range 2.7–6.1 years). The mean age at start of follow-up was 75.8 years (standard deviation ± 8.5 years). Characteristics of the study participants are presented in Table 1. The time from prostate cancer diagnosis to start of ADT ranged between 0 and 83 months: 71% of patients started ADT within 3 months of prostate cancer diagnosis; 13.5% of patients started ADT between 3 and 12 months of prostate cancer diagnosis; 15.5% of patients started ADT more than 12 months after prostate cancer diagnosis. The median (interquartile range 1–3) number of days of watchful waiting time from prostate cancer diagnosis to start of ADT where 41 (21–103) days. There were no major differences between ADT exposed and unexposed with regard to marital status or educational level. After the predetermined exclusion of the first year of follow-up, 270 new diagnoses of esophageal or gastric cancer were identified in the cohort.

**Table 1:** Characteristics of men exposed or not exposed to androgen deprivation therapy (ADT) in Prostate Cancer data Base Sweden.

	Non-ADT exposed (n, %)	ADT exposed (n, %)
Participants	84,174	17,560
Mean age at start of follow up (years)	75.3 ± 8.5	75.5 ± 8.4
**Marital status**		
Not married	8767 (10.1)	1769 (10.1)
Married or registered partnership	53,688 (61.8)	11,218 (63.9)
Separated (/registered partnership)	11,076 (12.8)	2358 (13.4)
Widower (/registered partnership)	10,510 (12.1)	2207 (12.6)
Missing values	133 (0.2)	8 (< 0.1)
**Education**		
Low (< 10 years)	38,489 (45.7)	8377 (47.7)
Middle (10–12 years)	28,715 (34.1)	5996 (34.1)
High (> 12 years)	15,154 (18.0)	2973 (16.9)
Missing values	1816 (2.2)	214 (1.2)

Continuous variables expressed as mean ± standard deviation. Categorical variables expressed as frequencies (n) with percentages (%).

### Risk of esophageal adenocarcinoma

During follow-up, 75 new cases of EAC were identified. Exposure to ADT was not followed by a decreased risk of EAC (HR 1.17 [95% CI 0.60–2.32]). Stratified into different forms of ADT, there was also no decrease in the risk of developing EAC (Table 2). Similarly, the sensitivity analysis pooling EAC and cardia GAC yielded no decreased risk for any ADT (HR 1.14 [95% CI 0.67–1.37]) or after stratifying into different forms of ADT (data not shown). Analysis of latency in an attempt to determine a time period effect in cancer development could not be performed due to a small number of cases (data not shown).

**Table 2:** Multivariable analysis of hazard ratio (HR) with 95% confidence interval (CI) of esophageal adenocarcinoma and esophageal squamous-cell carcinoma according to exposure to androgen deprivation therapy (ADT).

	Esophageal adenocarcinoma				Esophageal squamous-cell carcinoma			
	No	Yes	HR (95% CI) Crude	HR (95% CI) Adjusted	No	Yes	HR (95% CI) Crude	HR (95% CI) Adjusted
All men	101,659	75			101,708	26		
No ADT	84,112	62	1.00 (Reference)	Ref	84,152	22	Ref	Ref
All ADTs	17,547	13	1.25 (0.67–2.35)	1.17 (0.60–2.32)	17,556	4	1.03 (0.34–3.17)	0.99 (0.31–3.13)
AA	5300	2	0.64 (0.14–2.83)	0.65 (0.14–3.12)	5301	1	1.19 (0.13–10.69)	1.42 (0.14–13.89)
GnRH	2075	1	0.65 (0.08–5.67)	0.48 (0.05–4.42)	2075	1	3.46 (0.22–55.78)	3.29 (0.18–58.56)
GnRH + Flare	8245	8	1.62 (0.70–3.74)	1.55 (0.62–3.85)	8251	2	1.05 (0.22–4.98)	1.11 (0.23–5.47)
ORCH	1287	2	2.51 (0.42–15.09)	2.45 (0.38–15.70)	1289	0	–	–
TAB	640	0	–	–	640	0	–	–

Multivariable Cox proportional hazard model including marital status (not married, married, separated and widower) and educational level; low (less than 10 years), intermediate (10–12 years) and high educational level (> 12 years).

*AA* anti-androgens, *GnRH* gonadotropin releasing hormone, *GnRH + Flare* gonadotropin releasing hormone + flare protection with AA for a limited time (usually a month), *ORCH* orchiectomy, *TAB* total androgen blockade.

### Risk of cardia and non-cardia gastric adenocarcinoma

Among 169 cases of gastric adenocarcinoma identified during follow up, 50 were located in the cardia and 119 in the non-cardia. Use of ADT was associated with a decreased point estimate of any GAC (HR 0.64 [95% CI 0.37–1.09]), but it did not reach statistical significance. Subsite analysis yielded a statistically significant decrease in the risk of non-cardia GAC (HR 0.49 [95% CI 0.24–0.98]), but not for cardia GAC (HR 0.99 [95% CI 0.40–2.46]). When stratified into different forms of ADT, the point estimates indicated decreased HRs for non-cardia GAC, but did not reach statistical significance (Table 3). Examination of latency in GAC showed no time period effect (data not shown).

**Table 3:** Multivariable analysis of hazard ratio (HR) with 95% confidence interval (CI) of gastric adenocarcinoma according to exposure to androgen deprivation therapy (ADT).

	Total gastric adenocarcinoma				Cardia gastric adenocarcinoma				Non-cardia gastric adenocarcinoma			
	No	Yes	HR (95% CI) Crude	HR (95% CI) Adjusted	No	Yes	HR (95% CI) Crude	HR (95% CI) Adjusted	No	Yes	HR (95% CI) Crude	HR (95% CI) Adjusted
All men	101,565	169			101,684	50			101,615	119		
No ADT	84,024	150	1.00 (Reference)	Ref	84,132	42	Ref	Ref	84,066	108	Ref	Ref
All ADTs	17,541	19	0.64 (0.38–1.07)	0.64 (0.37–1.09)	17,552	8	0.95 (0.42–2.16)	0.99 (0.40–2.46)	17,549	11	0.52 (0.27–1.00)	0.49 (0.24–0.98)
AA	5298	4	0.41 (0.12–1.35)	0.33 (0.08–1.39)	5300	2	0.42 (0.05–3.33)	0.42 (0.05–3.88)	5300	2	0.41 (0.09–1.74)	0.24 (0.03–1.83)
GnRH	2074	2	0.39 (0.09–1.67)	0.42 (0.10–1.86)	2076	0	–	–	2074	2	0.50 (0.11–2.19)	0.52 (0.12–2.31)
GnRH + Flare	8243	10	0.73 (0.36–1.50)	0.74 (0.36–1.53)	8249	4	1.04 (0.35–3.13)	1.01 (0.30–3.37)	8247	6	0.59 (0.23–1.52)	0.60 (0.23–1.58)
ORCH	1287	2	1.16 (0.24–5.62)	1.04 (0.21–5.12)	1288	1	–	–	1288	1	0.60 (0.07–4.91)	0.52 (0.06–4.51)
TAB	639	1	4.00 (0.25–63.95)	5.17 (0.32–84.75)	639	1	–	–	640	0	–	–

Multivariable Cox proportional hazard model including marital status (not married, married, separated and widower) and educational level; low (less than 10 years), intermediate (10–12 years) and high educational level (> 12 years).

*AA* anti-androgens, *GnRH* gonadotropin releasing hormone, *GnRH + Flare* gonadotropin releasing hormone + flare protection with AA for a limited time (usually a month), *ORCH* orchiectomy, *TAB* total androgen blockade.

### Risk of esophageal squamous-cell carcinoma

During follow-up, 26 cases of ESCC were identified. Exposure to ADTs did not influence the risk of ESCC (HR 0.99 [95% CI 0.31–3.13]). Stratified analyses of different forms of ADT showed no associations (Table 2). Examination of latency effects was not performed due to a small number of cases (data not shown).

## Discussion

This study indicated that androgen deprivation therapy decreases the risk of non-cardia gastric adenocarcinoma, while no risk reduction was found for cardia gastric adenocarcinoma, esophageal adenocarcinoma, or esophageal squamous-cell carcinoma.

There are some strengths with this study. The population-based cohort design linking the NPCR to nationwide, comprehensive and high-quality health data registers yielded a large number of person-years at risk, a close to complete follow-up, and counteracted selection bias. As an evolution compared with previous work from our group on this topic^28^, this study made use of data on the type of ADT prescribed, however, this improvement was hampered by the small number of cases in each group as a result of the sub-group analysis. Moreover, cardia and non-cardia GAC were analyzed separately, which is relevant because of different etiologies. Several weaknesses need to be highlighted. Due to the relatively short time that has passed since the inception of the Prescribed Drug Register in 2005 and the low incidence of these cancers in Sweden, the follow-up was short, perhaps too short to capture a biologically relevant duration of exposure, and the statistical power was limited. The limited sample size also impeded the sub-group analyses of different forms of ADT. Our main finding of a reduced risk of non-cardia GAC could be due to a type I error i.e. a chance finding because there were a number of sub-group analysis conducted based across cancer sites and histological sub-types. However, that interpretation is opposed by the fact that the reduced risk of non-cardia GAC was our main hypothesis which was formulated à priori and was based on previously published results. Another limitation is the lack of data on potential confounders such as obesity, tobacco smoking, diet and other lifestyle-related risk factors as well as heredity factors as these are associated with both developing prostate cancer and esophageal or gastric cancer^29^. On the other hand, marital status, residential area and educational level were adjusted for. A further limit to the generalizability of this study is that since the study period ended in 2012, more recent forms of ADT, such as GnRH antagonists, are not included in the exposures and therefore treatments of prostate cancer patients.

In this study, we opted to employ a time-constant rather than a time-dependent (time-varying) model. Our hypothesis of a time-constant effect was tested and passed the Schoenfeld residuals test prior to running the model. A further limitation to this study and the main reason why we did not consider the exposure variable as time-dependent is that we only had information about the first prescription of ADT and defined our exposure group accordingly. Hence, we treated ADT as a time-constant exposure and made the assumption, based on previous research (see below), that patients have a high adherence to treatment. The time-constant approach allowed us to use Cox modeling as it will generate very similar results to a Poisson model in this context but is not as time-consuming. In addition, we decided to use a control group from the general population as opposed to or including a control group of prostate cancer patients that were not exposed to ADT. Using a control group of prostate cancer patients introduces different problems such as selection biases or confounding by indication. Moreover, including prostate cancer patients as both cases and controls introduces difficulties in generalizing the results of the study to other populations. Results could also have been difficult to interpret if we, à priori, had included prostate cancer patients as both cases and controls as well as controls from the general population and if the study had yielded opposing results.

This study found a reduced risk for non-cardia GAC among men exposed to ADT. A diagnosis of prostate cancer may lead to a positive change in lifestyle, potentially influencing the exposure to risk factors such as diet, tobacco smoking and obesity, lowering the risk of further malignancy. However, the strongly decreased risk estimate of non-cardia GAC in particular might not be explained only by lifestyle changes. Additionally, a previous study showing a reduced risk of EAC after a first diagnosis of prostate cancer found it unlikely that a change in lifestyle confounded their results because they observed, just like we did, that the risk of ESCC was not reduced^30^. Moreover, a study reported an overall increased risk of second primary tumors after a prostate cancer diagnosis rather than a decreased risk^31^. The high adherence to ADT in prostate cancer patients, i.e. the translation of prescribed to actually taken drug, has been validated^32^. Although age above 75 years and low-risk prostate cancer were associated with lower adherence, misclassification of exposure resulting from non-adherence to prescribed treatment should be randomly associated with the outcomes and dilute associations rather than explain them.

The lack of associations between ADT and risk of cardia GAC, EAC or ESCC is in support with a similar study^33^ but in conflict with others^30,34,35^. Many, including our group, have evaluated the effect of hormone replacement therapy (HRT) on the development of EAC and ESCC without being able to show any significant risk reductions^36,37^. However, a meta-analysis demonstrated a decreased risk of EAC in ever users of HRT^38^ which was confirmed in recent epidemiological research suggesting that HRT protects against the development of both EAC and ESCC^39^. Furthermore, in the development of EAC only breastfeeding has been shown to be associated with a decreased risk of EAC in a study of pooled data from several case–control studies^40^. Two separate meta-analysis^41,42^ confirmed this finding and suggested that HRT is protective whereas early menopause is a risk factor in the development of esophageal cancer. Further research is needed to provide the answer to the role of androgens and anti-androgens in the etiology of these tumors.

Several previous studies by our group^28,36,43^ and others^44–48^ have examined the association between sex hormones and GAC by evaluating the effects of HRT, anti-androgen therapy and anti-estrogen therapy. Our group has previously found a 13% (SIR 0.87, 95% CI 0.78–0.98) reduced risk of GAC in a national cohort study following prostate cancer patients defined as estrogen treated^28^. In another national cohort study, our group previously showed that use of the anti-estrogen tamoxifen increased the risk of GAC by 27% (SIR 1.27, 95% CI 1.03–1.57)^49^. The findings indicating that an anti-estrogen increases the risk of GAC has been corroborated by others^48^. However, a recent meta-analysis found no elevated risk of GC after tamoxifen therapy for breast cancer, regardless of the dose or the duration of the drug used and independent of the latency interval after breast cancer diagnosis^50^. In a nested case–control study, our group showed that women using HRT had a 52% (OR 0.48, 95% CI 0.29–0.79) reduced risk of GAC^36^. Results showing a protective effect of HRT on GAC risk have been corroborated by others. In a meta-analysis, higher number of fertile years and use of HRT was associated with a reduced risk and exposure to treatment with the anti-estrogen tamoxifen was linked to an increased risk of gastric cancer^51^. Furthermore, recent research suggests that HRT protects against the development of GAC^39^. However, there are studies in which no such association could be found^44^. Taken together, most previous studies are in agreement with the results of the present study and support the hypothesis that exposure to estrogen or anti-androgen effects may decrease the risk of non-cardia GAC.

The potential biological mechanisms behind the identified association remain unclear, but may include cell cycle and growth arrest as well as induced apoptosis^52^. It has been suggested that the possible protective effect of estrogen on gastric carcinogenesis is exerted through estrogen receptors^53^. Both androgen receptors and estrogen receptors modulate proliferation, migration and invasion of gastric cancer and are potential targets for future intervention^54^. Recent research has demonstrated that higher genetically predicted levels of follicle-stimulating hormone (FSH) were associated with increased risk of EAC and higher genetically predicted levels of luteinizing hormone (LH) were associated with decreased EAC risk^55^. Whether these associations are causal remains to be investigated. The first study of prediagnostic sex hormone levels in relation to EAC risk assessing both EAC and cardia GAC as a combined outcome found that higher concentrations of dehydroepiandrosterone (DHEA) and estradiol were associated with decreased EAC and cardia GAC risk but did not find any association with testosterone^56^. However, the second prospective study^57^ reported that higher concentrations of LH and testosterone were associated with decreased EAC risk in men, contrary to the original hypothesis that testosterone increases EAC risk. Further research is warranted to elucidate the associations of DHEA, LH, testosterone as well as estrogen and EAC risk.

If the results of the present study can be further verified, the potential future clinical implications may include the development of drugs which interact with androgen receptors in order to decrease the risk of non-cardia GAC especially in high risk individuals. However, further research to clarify biological mechanisms is warranted before any preventive measures could be attempted in high risk populations.

In conclusion, this Swedish nationwide population-based matched cohort study suggests that exposure to androgen deprivation therapy decreases the risk of non-cardia gastric adenocarcinoma, while no risk reduction was evident in the development of cardia gastric adenocarcinoma, esophageal adenocarcinoma or esophageal squamous-cell carcinoma in this study setting.

## Data Availability

All data and materials are available upon request.

## Code availability

All statistical analyses were performed using the statistical software Stata® (version 12.1; Stata Corporation, College Station, TX, USA). All coding is available upon request.
